# Isoprene Emission Influences the Proteomic Profile of Arabidopsis Plants under Well-Watered and Drought-Stress Conditions

**DOI:** 10.3390/ijms23073836

**Published:** 2022-03-30

**Authors:** Ilaria Mancini, Guido Domingo, Marcella Bracale, Francesco Loreto, Susanna Pollastri

**Affiliations:** 1 Department of Biotechnology and Life Science, University of Insubria, Via J.H. Dunant 3, 21100 Varese, Italy; imancini1@studenti.uninsubria.it (I.M.); marcella.bracale@uninsubria.it (M.B.); 2 Department of Biology, University of Naples Federico II, Via Cinthia, 80126 Naples, Italy; 3 Institute for Sustainable Plant Protection, National Research Council of Italy, Via Madonna del Piano 10, Sesto Fiorentino, 50019 Florence, Italy; susanna.pollastri@ipsp.cnr.it

**Keywords:** photosynthesis, chlorophyll fluorescence, *Arabidopsis thaliana*, proteomic, ABA

## Abstract

Isoprene is a small lipophilic molecule synthesized in plastids and abundantly released into the atmosphere. Isoprene-emitting plants are better protected against abiotic stresses, but the mechanism of action of isoprene is still under debate. In this study, we compared the physiological responses and proteomic profiles of Arabidopsis which express the isoprene synthase (ISPS) gene and emit isoprene with those of non-emitting plants under both drought-stress (DS) and well-watered (WW) conditions. We aimed to investigate whether isoprene-emitting plants displayed a different proteomic profile that is consistent with the metabolic changes already reported. Only ISPS DS plants were able to maintain the same photosynthesis and fresh weight of WW plants. LC–MS/MS-based proteomic analysis revealed changes in protein abundance that were dependent on the capacity for emitting isoprene in addition to those caused by the DS. The majority of the proteins changed in response to the interaction between DS and isoprene emission. These include proteins that are associated with the activation of secondary metabolisms leading to ABA, trehalose, and proline accumulations. Overall, our proteomic data suggest that isoprene exerts its protective mechanism at different levels: under drought stress, isoprene affects the abundance of chloroplast proteins, confirming a strong direct or indirect antioxidant action and also modulates signaling and hormone pathways, especially those controlling ABA synthesis. Unexpectedly, isoprene also alters membrane trafficking.

## 1. Introduction

Plants have developed many different defense systems and compounds to protect themselves against unfavorable environmental conditions [1,2]. Isoprenoids are a major class of volatile organic compounds (VOCs). Among them, isoprene (C_5_H_8_) is synthesized at the chloroplast level through the photosynthesis-dependent 2-C-methyl-d-erythritol 4-phosphate (MEP) pathway [2,3] and is a costly compound in terms of carbon and energy. For example, it is estimated that 0.5–2% of photosynthetic carbon is re-emitted in the atmosphere as isoprene [4].

Not all plants can emit measurable rates of isoprene (around 20% worldwide according to [5]). Several lines of evidence indicate that isoprene emitters are better protected against thermal, drought, and oxidative stresses [6,7,8,9,10,11], although isoprene synthesis and emission are not necessarily required for high rates of photosynthesis and woody biomass production, even under stress conditions [12].

The capacity to preserve high photosynthetic rates in adverse conditions is a desirable trait in a warming and drying world because of climate change influence. Many studies have been performed to understand how isoprene exerts its protective action, which seems to rely on a combination of effects. Thylakoid membranes of isoprene-emitting plants are more resistant to denaturation and maintain the same stiffness, fluidity, and functionality at rising temperatures compared with non-emitting plants [8,13]. This effect may depend on the chemical and physical properties of isoprene. The lipophilic properties of isoprene may strengthen the lipid layer of membranes [2,14,15]. Isoprene’s conjugated double bonds allow this molecule to react and scavenge many reactive and dangerous chemical species [11]. The low evaporation temperature (34 °C) may allow isoprene to remove heat from membranes [16].

Vickers et al. claimed that isoprene effects may all converge toward a protective function of the photosynthetic apparatus from generalist stresses [11]. However, this view is possibly challenged by a concentration of isoprene that is too low to effectively carry out any antioxidant action or to have other membrane protection properties [17]. Moreover, growing evidence of the reprogramming of the transcriptome, proteome, and metabolome in isoprene-emitting plants implies a much wider action of isoprene [17,18,19,20,21,22,23,24]. Very recently, the role of isoprene as a signaling and priming molecule has also emerged [18,25,26].

To further help gain an in-depth understanding of the molecular mechanisms underlying the effects of isoprene, we screened and identified changes in the proteomes of *Arabidopsis thaliana* plants that do not emit (wild type) or emit isoprene after the insertion of *Eucalyptus globulus* isoprene synthase (ISPS) under both control and drought-stress conditions. Transcriptomics analysis showed that many pathways are induced in these transgenic isoprene-emitting Arabidopsis lines [18]. Our working hypothesis was that proteins assembled by genes with transcripts that were altered by the insertion of ISPS should consistently change their accumulation patterns. However, due to post-transcriptional modification, translational regulation, and protein degradation, mRNA expression levels may not fully predict the corresponding protein abundance, and proteomics research can provide new and useful information that complements the transcriptomics results. Here, proteome analysis indicates specific and distinct changes associated with isoprene presence and metabolite distribution in well-watered and drought-stressed plants. The results provide useful information for further research elucidating isoprene’s mechanism(s) of action defending plants against stresses.

## 2. Results

### 2.1. Physiological Data

**Figure 1:**
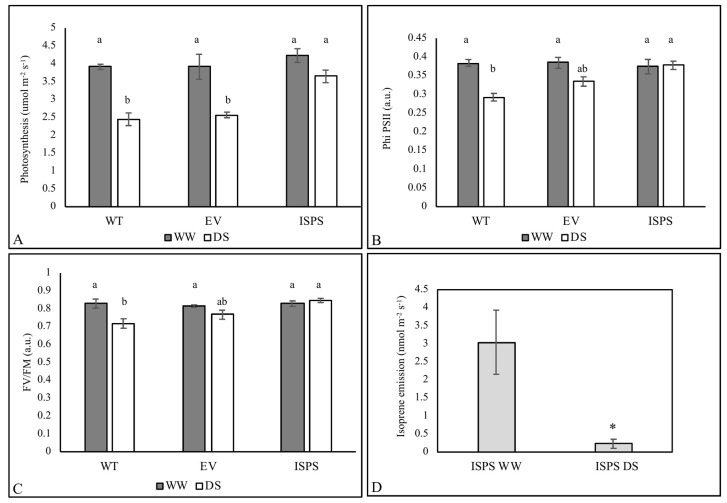
Photosynthesis (An) (**A**), PSII quantum yield (ΦPSII) (**B**), maximum quantum efficiency of photosystem II (Fv/FM) (**C**) of wild-type (WT), empty vector (EV), and isoprene-emitting (ISPS) plants in well-watered (WW, grey bars) and water-stressed (DS, white bars) conditions. Isoprene emission (**D**) of ISPS plants measured in well-watered and water stressed conditions. Means + SE (n = 5) are shown. One-way ANOVA followed by Tukey’s test was performed to statistically separate means (**A**–**C**). Means significantly different (*p* < 0.05) are represented by different letters (a, b, c). Student t test was applied to compare the isoprene emission in isoprene emitting plants in WW and WS conditions (* *p* < 0.05).

All plants showed the same photosynthesis (An) in well-watered (WW) conditions, whereas under drought stress (DS) An of the isoprene-emitting (ISPS) genotype was significantly less affected than in WT and EV plants (Figure 1A). Stomatal conductance (gs) was similar among plants and treatments in ISPS and EV plants. Drought-stressed WT plants showed the lowest gs values, although the differences were not statistically significant even in this case (Figure S1A). Chlorophyll fluorescence parameters revealed a similar pattern to An. ΦPSII and Fv/Fm were similar in the three WW lines and were significantly reduced only in lines that do not emit isoprene under DS (Figure 1B,C). The non-photochemical quenching (NPQ) of fluorescence was statistically similar in all the plants and treatments, with the lowest values recorded in ISPS plants (Figure S1B). Being significantly lower in DS than in WW conditions, Isoprene emission was measured only in ISPS plants (Figure 1D).

The fresh weight was significantly reduced in DS compared with WW conditions only in non-emitting plants (WT, EV) (Figure S1D). However, the reduction of dry weight and RWC under DS conditions was not significant in any plant (Figure S1E).

### 2.2. Overview of Proteomic Profiles

To highlight the molecular mechanisms underlying the different responses of isoprene-emitting and non-emitting plants under drought stress, we performed quantitative proteomic analysis of ISPS and WT plants only, since EV showed the same physiological responses as WT.

A total of 2625 proteins were identified and quantified. Detailed information of all proteins, including ID, intensity values, and FASTA headers is provided in Table S1. The whole-protein dataset was successfully mapped with UniProtKB ID. According to the one-way ANOVA consistency test (FDR < 0.01), 20% of the whole-protein dataset (528 proteins) were differentially abundant proteins (DAPs; Table S2). A post hoc Tukey’s test identified 275 DAPs (101 of which accumulated and 174 decreased) in the WT DS vs. WT WW comparison; 271 DAPs (123 accumulated and 144 decreased) in the ISPS DS vs. ISPS WW comparison; 284 DAPs (134 accumulated and 150 reduced) in the comparison between ISPS WW and WT WW; and 273 DAPs (161 accumulated and 112 reduced) when comparing the WW plants of ISPS and WT (Table S2).

**Figure 2:**
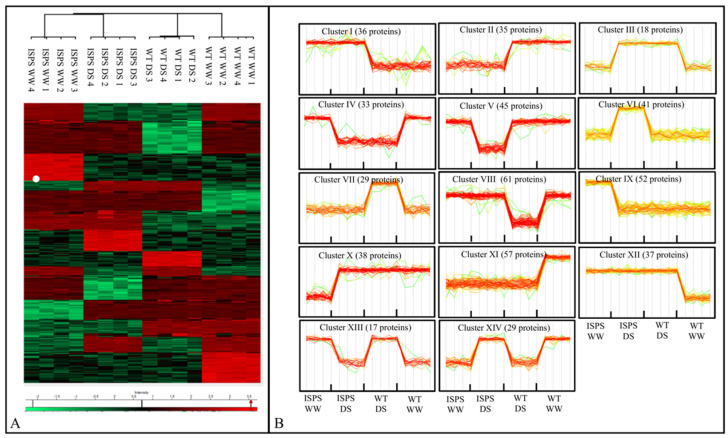
Heat map (**A**) and hierarchical clustering (**B**) of differentially abundant proteins (DAPs) according to one-way ANOVA analysis of wild-type (WT) and isoprene-emitting (ISPS) plants under well-watered (WW) and water stressed (WS) conditions. Heat map colours are based on the combined Z-scored (log_2_) LFQ values. Green and red shades correspond to proteins that accumulated less and more, respectively.

The heat map of the 528 DAPs revealed 14 distinct clusters of proteins changing in each condition (Figure 2A,B). Clusters I and II were composed of proteins that accumulated or decreased, respectively, in ISPS plants compared with WT plants, regardless of the growth condition. Cluster III and IV consisted of DAPs altered by DS, regardless of the genotype. The remaining clusters (V–XIV) consisted of DAPs differently modulated by DS, depending on their genetic background. DAPs belonging to clusters V and VI were affected by drought in the ISPS plants only; clusters VII and VIII were DAPs affected by drought solely in WT plants.

Correlation coefficients of the 16 samples (four replicates × four groups) showed high repeatability of the proteomic data. Moreover, the four experimental conditions were separated by the PCA score plot, and replicates within each group plotted very closely (Figure S2A). DS and WW plants were separated in PC1, while WT and ISPS plants were separated in PC2, accounting for 32.4% and 31.6% of the total variation, respectively. This indicates substantial and independent effects of both the capacity to emit isoprene and the stress treatment (Figure S2B).

### 2.3. Analysis of Proteomic Differences

A two-way ANOVA was performed on all 528 DAPs (FDR < 0.01) to assess any significant differences in protein abundance due to genotype (G), DS treatment (T), or the interaction between these two factors (I). Significant FDR values were selected for each variable (G, T, or I) and separately grouped in three protein lists: G-dependent, T-dependent, and I-dependent DAPs (Table S3A–C).

The single factors genotype and treatment significantly influenced the abundance of 78 and 55 DAPs, respectively (Table S3A,B). The 78 G-dependent DAPs (39 of which accumulated more in ISPS than in WT) represent the basic response of the Arabidopsis proteome to the acquired capacity to emit isoprene, regardless of plant growth conditions. This trend is described by Clusters I and II in Figure 2B.

On the other hand, the 55 T-dependent DAPs (19 of which accumulated in both genotypes exposed to DS more than in WW plants) responded to drought independently in their capacity to emit isoprene. Moreover, 67% of the T-dependent DAPs were reduced in their abundance, as expected for translation and protein synthesis under stress. The T-dependent DAP trend is displayed as Cluster III and IV (Figure 2B).

The interaction G x T (I) affected the abundance of 396 DAPs (Table S3C). The interaction between G and T, therefore, influences the proteome more than G or T singularly. The expression profiles of the I-dependent DAPs are represented by Clusters V–XIV (Figure 2B).

**Figure 3:**
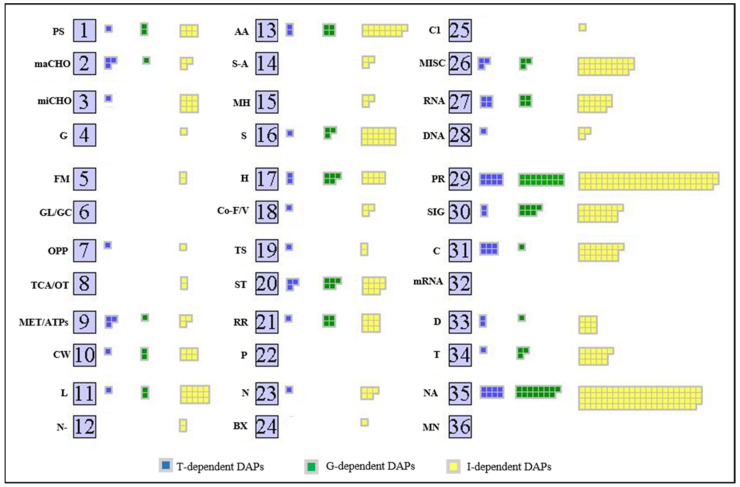
Functional classification of genotype (green squares, G), treatment (blue squares, T) and interaction-dependent (yellow squares, I) differentially accumulated proteins (DAPs) by MapMan Overview map. Each square represents a protein; The 36 BINS abbreviations: PS, photosynthesis; maCHO, major carbohydrate metabolism; miCHO, minor carbohydrate metabolism; G, glycolysis; FM, fermentation; GL/GC, gluconeogenesis/glyoxylate cycle; OPP, oxidative pentose phosphate; TCA/OT, tricarboxylic acid/organic acid transformations; MET/ATPs, mitochondrial electron transport/adenosine triphosphate; CW, cell wall; L, lipid metabolism; N-, nitrogen metabolism; AA, amino acid metabolism; S-A, sulphur assimilation; MH, metal handling; S, secondary metabolism; H, hormone metabolism; Co-F/V, co-factor and vitamin metabolism; TS, tetrapyrrole synthesis; ST, stress; RR, redox regulation; P, polyamine metabolism; N, nucleotide metabolism; BioDX, biodegradation of xenobiotics; C1, C1-metabolism; MISC, miscellaneous; RNA, ribonucleic acid; DNA, deoxyribonucleic acid; PR, protein; SIG, signalling; C, cell; mRNA, messenger RNA; D, development; T, transport; NA, not assigned; MN, mineral nutrition.

The MapMan annotation revealed that proteins of 16 out of 36 pathways were influenced by G, indicating a large reshaping of the proteome associated with the capacity to emit isoprene (Figure 3). For the G- and I-lists, functional classes included PROTEIN TRANSLATION, PROCESSING, AND DEGRADATION (20% and 18%, respectively), SIGNALLING (9% and 5%, respectively), TRANSPORT (4% and 4%, respectively), STRESS (6% and 3%, respectively), and HORMONE METABOLISM (6% and 2%, respectively). Functional classes in the T-list were PROTEIN TRANSLATION, PROCESSING, AND DEGRADATION (13%), CELL (10%), RNA (6 %), and STRESS (5%).

**Figure 4:**
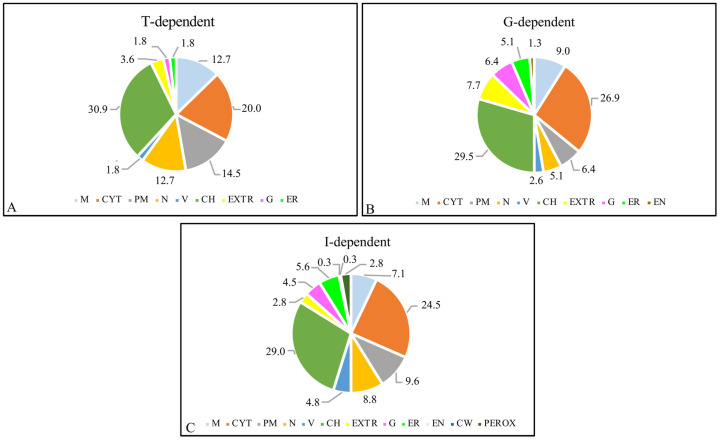
Subcellular localization of (**A**) treatment-dependent DAPs (T); (**B**) genotype-dependent DAPs (G); (**C**) Interaction-dependent DAPs (I), using the multiple marker abundance profiling method of the SUBA4 bioinformatic platform. Abbreviations: M, mitochondria; CYT, cytoplasm; PM, plasma membrane; N, nucleus; V, vacuole; CH, chloroplast; EXTR, extracellular region; G, golgi; ER, endoplasmic reticulum; EN, endosome; CW, cell wall; PEROX, peroxisome. Numbers in the pie charts represent percentages of DAPs belonging to each subcellular compartment with respect to total DAPs.

The predicted subcellular localization was consistent for the G, T, and I lists as proteins localized in chloroplasts were the most affected, ranging from 29 to 30%, followed by those located in cytosol (20–27% range). The genotype influenced a larger accumulation of proteins localized in GOLGI, ENDOPLASMIC RETICULUM, ENDOSOMES, and PEROXISOMES, compared with those subjected only to stress or to the interaction of the two factors (Figure 4, Table S4), suggesting possible sites of action of isoprene at the level of cell organization.

### 2.4. Free Proline, Trehalose, and ABA Accumulation

To provide indirect validation of the proteomic results, we measured the levels of free proline, trehalose, and ABA in WT and transgenic Arabidopsis (EV and ISPS plants) under WW and DS conditions.

**Figure 5:**
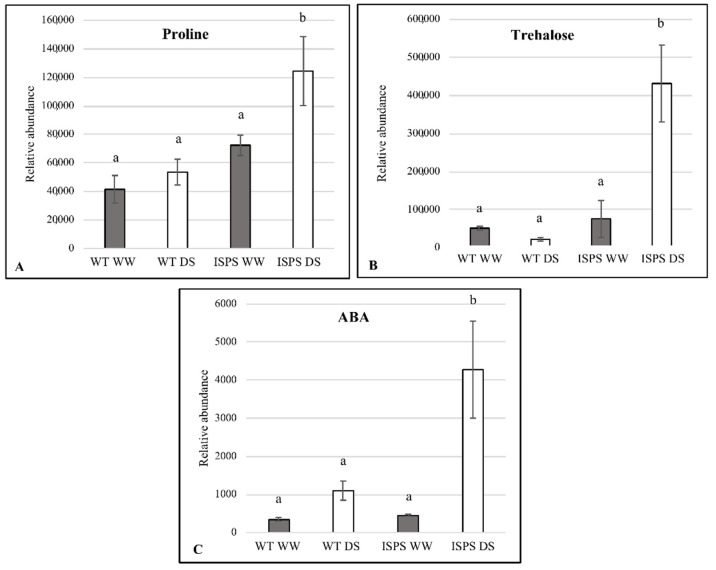
Proline (**A**), trehalose (**B**) and ABA (**C**) content in WT and ISPS genotypes under well-watered (grey column, WW) and water-stress (white column, DS) conditions. Different lowercase letters indicate significant differences at the *p* < 0.05 level using Kruskal-Wallis test and Dunn post-hoc comparisons method.

In both WT and EV lines, proline and trehalose did not accumulate significantly under DS compared with WW conditions. In ISPS transgenic Arabidopsis, in the absence of drought stress, both proline and trehalose contents were similar to those observed in WT, but they became significantly higher than in WT under DS conditions (Figure 5A,B). Similarly, there was no significant difference in ABA content between WT, EV, and ISPS plants under normal conditions. After the DS treatment, significantly higher levels of ABA were recorded only in ISPS, while in WT and EV drought-stressed plants, the ABA increase was not statistically significant (Figure 5C). The changes observed at the metabolite level fully mirrored those measured in the proteins involved in their biosynthesis (trehalose-phosphatase/synthase 7, Δ1-pyrroline-5-carboxylate synthetase 2, and urate oxidase).

## 3. Discussion

Isoprene-emitting plants can protect photosynthesis from several abiotic stress challenges [11,16]. Our physiological measurements confirmed such increased protection in isoprene-emitting Arabidopsis facing drought stress. We tried to provide further information on the protective mechanism of action of isoprene by examining the proteomic profile of non-emitting and isoprene-emitting plants. We showed that the induced capacity to emit isoprene causes a large reprogramming of the proteome and induces the synthesis of defense metabolites. In the following discussion, we analyze the main changing proteins (Table 1) and underlying biological processes that were affected by the genotype (G), the drought stress treatment (T), and the interaction between those two factors (I).

### 3.1. Genotype-Dependent Daps and Underlying Processes

Genotype-specific responses were shown in 78 DAPs (Table S3A). Differential accumulation of these proteins was exclusively caused by the acquired capacity to emit isoprene.

A large number of the DAPs were chloroplast-based and involved in chloroplast processes: photosynthesis, PSII assembly (ALB3, AT2G28800.4 [27]), the biosynthesis of xanthophylls (CYP97A3, AT1G31800.1) and flavonoids (chalcone-flavanone isomerase, AT1G53520.1), the control of superoxide anion radicals (Fe superoxide dismutase 1, AT4G25100.5, Dvořák et al. [28]), the photodamage repair cycle (FtsH11, AT5G53170.1, [29]), and chloroplast import and synthesis processes (HOP1, AT1G12270.1; S19, ATCG00820.1; RPOB, ATCG00190.1). These results confirm a positive correlation between isoprene emission and photosynthetic pigments [17,18,30,31] and might indicate a modified composition of the photosynthetic antenna in isoprene-emitting plants, allowing better light capture and better photoprotection of photosynthesis. This could explain the stability of PSII quantum yield and NPQ under stress conditions, observed here and often reported earlier [15]. It should also be observed that the proteins found as differentially abundant in our study are not coded by the genes reported by Zuo et al. [18] for ISPS transgenic Arabidopsis plants. This indicates an even more complex impact of isoprene than hitherto evident.

In naturally isoprene-emitting poplars, the synthesis of metabolites with a protective capacity is induced by the RNAi silencing of isoprene production [20,22]. This is accompanied by the upregulation of proteins in the pathways that control carotenoid and α-tocopherol biosynthesis [12]. These recent results indicate that isoprene acts as an intra- and inter-plant signaling-network coordinator, able to enhance plant performance against environmental cues through changes in gene expression and protein abundance [18,32]. Our proteomic results confirm this role and reveal new implications, showing that isoprene induces changes in proteins involved in both calcium (BON2, AT5G07300.1) and plant hormone signaling. Interestingly, we did not find isoprene-induced changes in proteins related to jasmonic acid, gibberellic acid, cytokinin, or oxylipins, as recently reported by other authors [32,33] and as expected given that isoprene influences senescence via cytokinin control [34]. Our analysis showed changes in: auxin transporter PIN3 (AT1G70940.1) [35,36]; mitogen-activated protein kinase 5 (MKK5, AT3G21220.1), which is involved in ABA regulation and phosphoribosyl anthranilate isomerase (AT5G05590.1), which catalyzes the third step in the biosynthetic pathway of tryptophan; EXORDIUM-like 4 protein (AT5G09440.1), with a role in the brassinosteroid-dependent regulation of plant growth and development [37].

In eukaryotic cells, endomembrane trafficking is vital for physiological processes, and in plants it is closely related to stress tolerance in order to meet the cell’s request for rapid molecular changes and to ensure the rapid delivery of stress-related cargo molecules [38]. The results of the proteomic analysis highlighted the accumulation in isoprene-emitting plants of the pivotal proteins involved in different steps of membrane trafficking, such as GET3A (AT1G01910.4), COG1 (AT5G16300.3), SNF7.1 (AT4G29160.3), CLUB/TRS130 (AT5G54440.1), and TRAPPC5 (AT5G58030.1).

### 3.2. Treatment-Dependent Daps and Underlying Processes

The proteomic analysis did not reveal significant variations in the abundance of the classical drought-related molecular chaperones and enzymes involved in drought signaling, ROS scavenging, and hormone synthesis, as reported elsewhere [39,40,41,42,43]. This may indicate that our plants experienced a mild stress compared to those activating the proteome in other experiments. However, some interesting features of plant drought response were highlighted by our proteomic approach.

The inhibition of metabolic processes is one of the primary detrimental effects of stresses. Accordingly, about two-thirds of the DAPs belonging to the T-list were less abundant in DS than in WW plants, both in the isoprene-emitting and non-emitting genotypes. Among these proteins, some participating in chlorophyll biosynthesis (CPP1, AT5g23040) and involved in PSII quantum efficiency (K^+^ efflux antiporter 3, AT4G04850.2) were identified. On the other hand, several chloroplast proteins are already known to be responsive to osmotic stress accumulated in DS plants. Among them, we highlight those proteins acting in the inositol signaling play, which has a crucial role in various aspects of plant stress adaptation (HEMA1, AT1G58290.1; Tetratricopeptide repeat-like superfamily protein, AT3G53560.1; inositol monophosphatase, AT4G05090.1) [44].

In addition to acting on chloroplast proteins, DS induced the accumulation of proteins known to be involved in the fine-tuning of Ca^2+^ and ROS to regulate stomatal closure (CML20, AT3G50360.1, [45]); protein turnover in response to abiotic stress (26S proteasome non-ATPase regulatory subunit 2 homolog B, AT4G28470.1, [46]); and the expression of downstream genes responsive to different abiotic stresses (ROF2, AT5G48570.1). Interestingly, the most reduced DAP in this list was ROF2 (AT5G48570.1), a negative regulator for the transcriptional activity of HSFs, which are key components of signal transduction that mediate the expression of several genes that are responsive to a variety of abiotic stresses [47,48].

Proteomic evidence seems also to indicate that DS-induced hormonal re-modulation involves brassinosteroid (BR) biosynthesis (3-oxo-5-alpha-steroid 4-dehydrogenase, AT5G16010.1) and auxin efflux and polar auxin transport (auxin transport protein, AT3G02260.1) [49]. BRs play a prominent role in controlling the hormonal balance in drought-adapted plant growth. An antagonistic role between ABA and BRs has been shown, but the complexity of BR-mediated responses to drought stress has not yet been fully understood [50,51]. The interdependent and often synergistic action of the auxin and BR pathways has been reported, occurring through both the transcriptional regulation of common target genes and the upstream connection involving calcium–calmodulin and phosphoinositide signaling [52,53].

### 3.3. Genotype–Treatment Interaction-Dependent Daps and Underlying Processes

In the I-list, 81 proteins were found to accumulate or be reduced in ISPS DS plants compared with all the other conditions tested (ISPS WW, WT DS, and WT WW). These proteins represent the specific impact of the isoprene–drought-stress interaction.

Osmotic stress triggers the accumulation of osmoprotectants that stabilize and protect biological structures from damage and may function as potent signaling molecules and ROS scavengers. Proteins that are known to be involved in the biosynthesis of trehalose (trehalose-phosphatase/synthase 7, AT1G06410.1) and proline Δ1-(pyrroline-5-carboxylate synthetase 2, AT3G55610.1) accumulated in the I-list only in ISPS DS plants. These two proteins are ABA-dependent [54,55]. Consistently, urate oxidase (AT2G26230.1), involved in ABA production, also accumulated only in ISPS DS plants.

To support these findings, we analyzed whether there was a variation in the accumulation of ABA, proline, and trehalose in isoprene-emitting and non-emitting lines. The three metabolites significantly increased in ISPS DS plants compared with all other treatments and conditions. This result is a robust orthogonal validation of the quality of the proteomic analysis. Moreover, the absence of physiological responses in WT and EV plants (both non-emitting) confirm that no transfection-dependent, unwanted alterations were found. Overall, these findings support the hypothesis that isoprene-emitting plants have a prompter response to water stress compared with non-emitting plants [32] and confirm that isoprene can prime defensive secondary metabolism [26], also inducing synthesis of osmoprotective compounds that protect plants under stress conditions.

The observed association of isoprene with ABA signaling is very interesting as ABA is formed by the oxidative cleavage of xanthophylls in the MEP pathway that also produces isoprene. Moreover, a correlation between ABA and isoprene was reported [56], and isoprene was hypothesized to interact with several hormones made by MEP, thus regulating leaf senescence [57]. Isoprene may simply proxy ABA, but it may also compete for substrates with ABA as both compounds are formed by the same methylerythritol phosphate (MEP) pathway in chloroplasts. This further supports the results indicating that ABA increases when isoprene is reduced by severe water stress [24]. In any case, our results strengthen the idea that isoprene is an important mediator of stress hormones, perhaps even deploying hormonal action by itself [26].

It is an established notion that increasing ABA content in leaves induces stomatal closure, thereby reducing water loss in drought-stressed leaves [58,59]. This is not the case with our plants where gs was never statistically different across samples and treatments, irrespective of ABA level. ABA conjugates (e.g., ABA–GE) are unable to affect stomata [60] and plants able to maintain their apoplastic pH unchanged are also poorly affected by ABA [61]. Causes that might uncouple ABA increase and gs reduction were reviewed by (Wilkinson & Davies 2002). Perhaps isoprene emission only proxies foliar ABA content, but this is not in the active form required for stomatal closure to occur or is not located at the guard cell where stomatal closure is actively controlled. ABA induces NO and H_2_O_2_ synthesis and all three cooperate to induce stomatal closure [62]. However, isoprene removes NO and ROS (reviewed by Pollastri et al. [26] and further discussed below) and this might also have lessened the stomatal response to increasing ABA in water-stressed ISPS plants. Finally, ABA is not the only factor involved in stomatal closure. Stomata may also close passively in response to the reduction of the vapor pressure difference between the leaf and the air [63]. As we kept the vapor pressure difference between leaf and air constant during foliar gas-exchange measurements, this might have also temporarily counteracted the stomatal closure otherwise occurring in drought-stressed plants.

Drought stress induces the production of ROS resulting from impaired efficient use of light energy by electron transport. The antioxidant action of isoprene was shown by early work [64], and a direct reaction between isoprene and singlet oxygen, in particular, was demonstrated [65]. Our proteomic characterization confirmed that isoprene modulates the oxidative load under DS through the accumulation of DHAR2 (AT1G75270.1), copper/zinc superoxide dismutase 3 (AT5G18100.2), uncoupling protein 5 (AT2G22500.1), PHOS32 (AT5G54430.1), TCP-1/cpn60 chaperonin (AT3G13470.1), 1-aminocyclopropane-1-carboxylate oxidase 3 (AT1G12010.1), and the reduction of PDIL2-2 (AT1G04980.1), SOUL heme-binding (AT3G10130.1), and thioredoxin (AT1G21350.3).

Plants must cope simultaneously with multiple stresses and elaborate stress-responsive networks with frequent cross-talk between metabolites that improves abiotic stress tolerance and disease resistance. Interestingly, seven defense-responsive proteins known to be involved in pathogen attack were found to be altered in ISPS DS plants: VAD1 (AT1G02120.1), PERK1 (AT3G24550.1), eukaryotic translation initiation factor isoform 4E (AT5G35620.1), dirigent-like protein 20 (AT1G55210.2), and PEN2 (AT2G44490.1) [66,67,68,69]. Moreover, the protein phosphatase 2C (AT4G33500.1), which negatively correlates with immunity to bacterial pathogens, was reduced. By contrast, MOS4 (AT3G18165.1), which is essential for plant innate immunity, and disease resistance protein RLM3 (AT4G16990.2), which is important for defense against necrotrophic fungi, both accumulated in ISPS DS and WW plants compared with WT. The activation of a large group of disease-protection-related proteins seems to indicate that, when elicited by abiotic stress, isoprene-emitting plants can also be primed against possible incoming biotic stresses [26], as further discussed below.

Interestingly, several I-dependent DAPs are similarly altered in DS plants and in isoprene-emitting WW plants. These are drought-related proteins the abundance of which is affected by isoprene, even under unstressed conditions. This subset includes chloroplast-related proteins (low PSII Accumulation protein, AT5G48790.1; FdC2, AT1G32550.1; ATPI, ATCG00150.1; and RPOC2, ATCG00170.1); proteins involved in all the different steps of membrane trafficking (among which are RABB1b, AT4G35860.1; CEF, AT3G44340.2; MKK2, AT4G29810.1; RER1B, AT2G21600.1; TRAPPC11, AT5G65950.1; and CRK7, AT4G23150.1); and in sugar-level modulation, ABA metabolism and action (KIN10, AT3G01090.3; NDR1/HIN1-like 1, AT3G11660.1; AOX, AT3G22370.1; and CPK29, AT1G76040.2). We again note that ABA is slightly (but not significantly, at least with the low number of replicates used in our experiment) higher in WW ISPS compared with WW WT, again confirming the proposed direct control of isoprene by ABA proposed earlier in this paper, and experimentally reported by Barta and Loreto [56].

## 4. Materials and Methods

### 4.1. Plant Material, Growth Conditions, and Drought-Stress Treatment

The study was performed using three different *Arabidopsis thaliana* (Col-0) genotypes: the non-emitting wild type (WT), the empty-vector line EV-B3 (EV), and the isoprene-emitting line C4, where the isoprene synthase gene from *Eucalyptus globulus* was inserted (ISPS). A detailed description of the transgenic Arabidopsis lines, kindly provided by Prof Thomas Sharkey, may be found in Zuo et al. [18]. Seeds were sown in moistened peat pellets (size 41 mm, pH 5.3—Jiffy Products), kept at 4 °C for 3 days, and then transferred to a growth room with the following conditions: photoperiod of 12 h with a light intensity of 150 µmol photons m^−2^ s^−1^ (fluorescent light) and temperature of 22 °C. During the night, the temperature was reduced to 20 °C. Normal tap water was used for all the experiments.

Six-week-old plants were divided into two groups, with one subjected to drought stress (DS) and the other kept in well-watered conditions (WW) and used as a control. The DS group was maintained in controlled DS by daily weighing of the pellets and keeping the soil moisture at 30% of field capacity. The DS condition was reached after 5 days and plants were kept in this condition for 5 further days. At this point, plants were subjected to the following measurements and analyses.

### 4.2. Leaf Gas–Exchange and Chlorophyll Fluorescence Measurements

A Li-Cor 6400-XT portable photosynthesis system (Licor, Inc., Lincoln, NE, USA) was used to measure photosynthesis (An, µmol m^−2^ s^−1^) and stomatal conductance (gs, mmol m^−2^ s^−1^) in five plants for each genotype and treatment. Before measuring, leaves were allowed to reach steady-state An and gs inside the 2 cm^2^ cuvette under 400 ppm of CO_2_, 200 µmol m^−2^ s^−1^ of light intensity, and a leaf temperature of 22 °C. Chlorophyll fluorescence parameters were also measured at this stage, namely the maximum quantum efficiency of photosystem II (PSII) by the ratio between variable and maximal fluorescence in darkened leaves (Fv/Fm); the PSII quantum yield (ΦPSII = Fm’−Fs/Fm’) where Fm’ is the maximal fluorescence and Fs is the steady-state fluorescence in light-adapted leaves; the non-photochemical quenching (NPQ = Fm−Fm′/Fm) were Fm′ is the maximal fluorescence in light-adapted (1000 µmol m^−2^ s^−1^) leaves [70].

### 4.3. Biomass and Leaf Relative Water Content and Measurements

The rosette leaves of five Arabidopsis plants for each genotype and treatment were harvested and their fresh weight recorded. The leaf relative water content (RWC, %) was estimated as described previously [71]. In detail, harvested leaves were placed in a jar with distilled water overnight after which the leaves were wiped, and the turgid weight was measured. Then the samples were dried in an oven at 60 °C until the dry weight (DW) was stable and could be measured.

Isoprene sampling and quantification was performed on fully expanded leaves enclosed inside the cuvette. A total of 5 L of air was collected from the cuvette outflow using a mass flow pump set at a 200 mL min^−1^ rate (AC Buck Inc., Orlando, FL, USA) and a cartridge filled with absorbents (30 mg each of Carbosieve X and Carbosieve B, Supelco, Bellefonte, PA, USA). Isoprene from emitting lines was quantified using an Agilent 5975 gas-chromatograph–mass-spectrometer (GC–MS) system fitted with an HP-INNOWax (50 m length, 0.2 mm ID, 0.4 µm film) column. Thermal desorption was executed by a Twister^®^ multipurpose autosampler and TD unit (Gerstel Technologies, Mülheim an der Ruhr, Germany) with an e-Trap cryofocussing system (Chromtech, Idstein, Germany). The GC separation program was 40 °C for 1 min, then ramping to 110 °C at 5 °C min^−1^ and remaining stable at 110 °C for 10 min and then increased to 260 °C at 30 °C min^−1^ and maintained at the final temperature for 2 min.

### 4.4. Protein Extraction

Proteins were extracted from 150 mg of leaf samples by the SDS/phenol method [72]. Briefly, leaves were ground in liquid nitrogen and homogenized with extraction buffer (0.15 M TRIS—HCl, pH 8.8, SDS 1%, 1 mM EDTA, 0.1 M DTT, 2 mM PMSF, 0.1 mg/mL Pefabloc, Protease Inhibitor 1:1000). After centrifugation, a 1:1 volume of phenol saturated with 0.1 M TRIS-HCl was added to the supernatant phase. The phenolic phase was collected and overnight precipitated with four volumes of 0.1 M ammonium acetate in methanol at −20 °C. The precipitate obtained by centrifugation at 15,000× *g* for 10 min at 4 °C was washed twice with cold 0.1 M ammonium acetate and finally with cold 80% acetone. The pellet was dried and resuspended in 250 μL of SDS-lysis buffer (20% *w*/*w* SDS, 0.25 M Tris/HCl pH 7.5, 100 mM DTT). Protein concentrations were quantified using the 2-D Quant-kit (GE Healthcare). Four independent biological replicates were analyzed for each sample.

### 4.5. Trypsin Digestion

Protein extracts were digested using the filter-aided sample preparation (FASP) protocol [73]. Briefly, protein extracts were heated for 5 min at 95 °C, diluted 10 times with UA buffer (8 M urea in 100 mM Tris-HCl, pH 8.0), and transferred to the YM-30 micron filter units (Millipore, Darmstadt, Germany). The denaturation buffer was replaced by washing 3 times with UA buffer and proteins were alkylated using 50 mM iodoacetamide in UA for 15 min at room temperature in the dark. The excess alkylation reagents were eliminated by washing 4 times with ABC buffer (50 mM NH_4_HCO_3_). Proteins were digested overnight at 37 °C with trypsin in ABC buffer at an enzyme-to-substrate of 1:100 (*w*/*w*) ratio. The digested peptides were eluted by centrifugation. Peptide concentrations were measured spectrophotometrically, assuming that a solution of proteins with a concentration of 1 mg mL^−1^ determines an absorbance of 1.1 at 280 nm. The peptides were finally desalted onto C18 Oasis-HLB cartridges and dried down for further analysis.

### 4.6. LC–MS/MS Analysis and Elaboration of Raw Data

The peptides (1 μg) were analyzed as described in [74]. Mass spectrometry analysis was performed on a QExactive mass spectrometer coupled to a nano EasyLC 1000 (Thermo Fisher Scientific Inc., Waltham, MA, USA). The solvent composition was 0.1% formic acid and 0.1% formic acid plus 99.9% acetonitrile, respectively, for channels A and B. For each sample, 4 μL of peptides were injected on a self-made column (75 μm × 150 mm) packed with reverse-phase C18 material (ReproSil-Pur 120 C18-AQ, 1.9 μm; Dr. Maisch GmbH, Ammerbuch, Germany). The flow rate was 300 nL/min by a gradient from 2 to 35% B in 80 min, 47% B in 4 min, and 98% B in 4 min. The order of the sample acquisition was randomized. The mass spectrometer was operated in data-dependent mode (DDA), full-scan MS spectra were acquired between 300 and 1700 m/z, and the twelve most intense signals per cycle were fragmented. HCD spectra were acquired at a resolution of 35,000 using a normalized collision energy of 25 and a maximum injection time of 120 ms. The automatic gain control (AGC) was set for 50,000 ions. Charge-state screening was enabled, and singly assigned and unassigned charge states were rejected. Precursor masses selected from the previous measurements were excluded for 30 s, and 10 ppm was used as an exclusion window. The samples were acquired using internal lock mass calibration on m/z 371.1010 and 445.1200.

Raw data were searched against the *Arabidopsis thaliana* Uniprot protein database (version 2019-01, 76,141 entries) with the MaxQuant program (v.1.5.3.3).

Protein identification was performed following these criteria: two missed cleavages; fixed modification of cysteine (carbamidomethylation); variable modifications of methionine (oxidation); and phosphorylation of serine, threonine, and tyrosine, with a minimum peptide length of 6 amino acids, precursor mass tolerance of 4.5 ppm for the main search. Label-free quantification (LFQ), “match between runs” (time window of 0.7 min), and target-decoy search strategy (revert mode) options were enabled. A false discovery rate (FDR) of 1% was accepted for both peptide and protein identification.

The raw data obtained as output from MaxQuant (“ProteinGroups” files) were initially processed using the Perseus software platform (http://www.perseus-framework.org, accessed on 1 March 2021). Incorrect identifications (“reverse”, “one site”, and “contaminant” hits) and inconsistent identifications were filtered out.

Imputation of the missing value was performed with an in-house tool. Missing values were estimated from the dataset based on two criteria for each sample, depending on whether one or more missing values were observed for each entry: when two or three values were available, the missing value was set to a random value within an interval of one-fourth of the entire sample standard deviation centered on the entry average. When only one or no values were available, random values within an interval of one-fourth of the standard deviation of all sample values centered on the global minimum value of all samples in the dataset were imputed. The minimum dataset value and sample standard deviations were determined once before any imputation and applied to all subsequent imputations to avoid drift.

For the quantitative proteome analyses, the filtered data were processed with the Perseus software platform (http://www.perseus-framework.org, accessed on 1 March 2021). Log_2_ transformed LFQ intensities of protein group intensities were centered by subtracting the median of the entire set of protein-group LFQ intensities per sample (column).

The mass spectrometry proteomics data were deposited in the ProteomeXchange Consortium via the PRIDE partner repository [75] with the dataset identifier PXD025069 using the following reviewer account details: Username: reviewer_pxd025069@ebi.ac.uk; Password: ok3Yiae5.

### 4.7. Quantification of Free-Proline Content

Free proline was extracted from lyophilized leaves (0.2 g FW per genotype per condition in four biological replicates), grounded, and resuspended in 1 mL of 0.1% (*v*/*v*) formic acid (FA) in water/methanol (MeOH) (50:50). After 4h mixing in the dark, the mixture was centrifuged (15 min, 13.000 rpm) and the supernatant collected. Proline standard solution (1mM in 0.1% FA and 50% MeOH) was prepared from 10 mM stock solution in distilled water and used for the calibration (range 2–15 µM). The HPLC analyses were performed in a Finnigan Surveyor MS plus HPLC system (Thermo Electron Corporation, CA, USA). For proline quantitation, separation was achieved using the C18 column (ACQUITY UPLC Peptide BEH C18 Column, 300Å, 1.7 µm, 2.1 mm × 150 mm). The mobile phase was composed of (A) water with 0.1% (*v*/*v*) formic acid and (B) methanol/water (50:50) plus 0.1% (*v*/*v*) formic acid with a flow rate 150 µL/min; gradient 0–3.0 min/2% (*v*/*v*) B, 3–16 min/2–50% (*v*/*v*) B. For the mass spectrometry quantification, a Finnigan LXQ linear ion trap mass spectrometer, equipped with an ESI ion source (Thermo Electron Corporation, CA, USA) was used. The analyses were performed in positive (spray voltage 4.5 kV, capillary temperature 270 °C) and in the multiple-reaction monitoring (MRM) mode. The optimization of collision energy for each substance, the tuning parameters, and the choice of fragments to confirm the identity of target compounds (proline) were conducted in continuous flow mode by using a standard solution at a concentration of 5 μM. MRM acquisition was accomplished monitoring the 116/70 transition. Free-proline contents were expressed as relative intensities among samples.

### 4.8. Determination of ABA Content

The abscisic acid (ABA) content was determined as described by Pan et al. [76], with three biological repetitions. Briefly, 20 mg of freeze-dried leaf material was ground into powder in liquid nitrogen using a mortar and pestle and extracted by adding 800 μL of 2-propanol/H_2_O/concentrated HCl (2:1:0.002, *v*/*v*/*v*). After shaking for 30 min at 4 °C, 1 mL of dichloromethane was added to each sample. After a further shaking for 30 min at 4 °C, samples were centrifuged at 13,000× *g* for 5 min at 4 °C and the lower phase was collected and concentrated (not completely dry) under nitrogen flow. Samples were then re-dissolved in 0.05 mL of methanol/H_2_O/formic acid (2:1:0.1, *v*/*v*/*v*). Separations were performed on an Acclaim RSLC 120 C8 column (Thermo Scientific; 2.2 µm, 120 Å, 2.1 × 100 mm) at 25 °C on a gradient elution at the flow rate of 0.2 mL min^−1^, using a Finnigan Surveyor MS plus HPLC system (Thermo Electron Corporation, Santa Clara, CA, USA). The mobile phase was composed of water with 0.1% (*v*/*v*) formic acid (solvent A), and methanol plus 0.1% (*v*/*v*) formic acid (solvent B). The gradient elution program was: 0–3.0 min/5% (*v*/*v*) B, 3–20 min/5–50% (*v*/*v*) B. A Finnigan LXQ linear ion trap mass spectrometer equipped with an ESI ion source (Thermo Electron Corporation, CA, USA) was used for ABA quantification. The analyses were performed in the negative (spray voltage 2.5 kV, capillary temperature 250 °C) and in multiple-reaction monitoring (MRM) mode. The optimization of collision energy for each substance, the tuning parameters, and the choice of fragments to confirm the identity of target compounds (ABA) were conducted in continuous flow mode by using a standard solution at a concentration of 0.01 mg mL^−1^. MRM acquisition was accomplished monitoring the 263/153 transition. ABA contents were expressed as relative intensities among samples.

### 4.9. Trehalose Determination

Lyophilized leaves (20 mg) in three biological replicates were ground into powder in liquid nitrogen using a mortar and pestle. Trehalose was extracted in 1 mL of water/ethanol (50:50) using a thermal mixer at 60 °C and 250 rpm for one hour. After centrifugation at 13,000× *g* for 5 min, the supernatant was collected and dried in a speedVac vacuum evaporator at room temperature.

Monosaccharide permethylation was performed according to the procedure described by [77], with minor modifications. Briefly, the dried samples were dissolved in 1 mL of DMSO, introduced into a conical glass vial, and 50 μL of water were added, as suggested by Ciucanu and Costello [78]. After the addition of 40 mg of finely powdered NaOH, samples were stirred vigorously at room temperature and then 80 μL methyl iodide was added. The mixture was stirred at room temperature for 10 min. Permethylated monosaccharides were extracted 2 times using 1000 μL of dichloromethane. The dichloromethane phases were then washed with 1000 μL of water at least 2 times to remove any residual salts. The organic phases containing the permethylated monosaccharides were dried under nitrogen flow and reconstituted in 50 μL of 0.1% (*v*/*v*) formic acid (FA) in water/methanol (MeOH) (50:50).

Separations were performed on an Acclaim RSLC 120 C8 column (Thermo Scientific; 2.2 µm, 120 Å, 2.1 × 100 mm) at 25 °C on a gradient elution at the flow rate of 0.2 mL/min using a Finnigan Surveyor MS plus HPLC system (Thermo Fisher Scientific, Whaltam, MA, USA). The mobile phase was composed of water with 0.1% (*v*/*v*) FA (solvent A), and methanol plus 0.1% (*v*/*v*) FA (solvent B). The gradient elution program was: 0–3.0 min/5% (*v*/*v*) B, 3–25 min/5–50% (*v*/*v*) B. A Finnigan LXQ linear ion trap mass spectrometer equipped with an ESI ion source (Thermo Fisher Scientific, Whaltam, MA, USA) was used for trehalose quantification. The analyses were performed in the negative (spray voltage 2.5 kV, capillary temperature 250 °C) and in MRM mode. The optimization of collision energy for each substance, the tuning parameters, and the choice of fragments to confirm the identity of permethylated trehalose were conducted in continuous flow mode by using a standard solution at a concentration of 0.001 mg/mL. MRM acquisition was accomplished monitoring the 477/259 transition. Trehalose contents were expressed as relative intensities among samples.

### 4.10. Statistical Analysis

Physiological data are shown as means ± standard errors (SEs). Data were analyzed using the Shapiro–Wilk test to determine the normality of the distribution and a one-way analysis of variance (ANOVA), with a critical *p*-value set at 0.05. All analyses were performed using Sigma Plot software (Systat Software Inc., San Jose, CA, USA).

To analyze the proteins’ changes in relative abundance between analytical groups we performed a statistical analysis on Perseus software (Max-Planck-Institute of Biochemistry, Martinsried, Germany; version 1.5.8.5). Transformed, centered, and normalized Log_2_ LFQ data were subjected to one-way ANOVA based on multiple-sample tests; an FDR cut-off of 0.01, based on the Benjamini–Hochberg correction was used for truncation. One-way ANOVA was coupled with Tukey’s test (FDR < 0.01) to extrapolate statistically significant comparisons. Two-way ANOVA was then performed on significant data to directly link the influence of genotype and treatment (drought stress) or the interaction between them.

The protein-fold change (FC) ratio, expressed as Log_2_FC, was defined as the Log_2_ of protein abundance in one biological condition minus Log_2_ of protein abundance in another biological condition.

The Perseus software was also used for principal component analysis (PCA) and plot scattering to assess the quality of our datasets. To assess the statistical significance of proline quantification, the Kruskal–Wallis test was applied and coupled with the Dunn post hoc comparisons method (*p* < 0.05).

### 4.11. Downstream Bioinformatics Analysis

Functional annotation and metabolic pathway analysis were performed with MapMan 3.6.0RC1 software [79] using the *Arabidopsis thaliana* ISOFORM_TAIR10_2012 protein database as background.

Perseus software was used to build heat maps and hierarchical clustering [80]. Hierarchical clustering of significantly changing proteins was performed using the Z-score calculation on Log2 intensity values with the following settings: row, column distance calculated using the Euclidean algorithm; row, column linkage—complete.

The potential subcellular localization of all the DAPs was analyzed using the SUBA4 bioinformatics platform (https://suba.live/, accessed on 30 March 2021 [81]).

One-way ANOVA of repeated measures followed Tukey’s post hoc test was used to verify ABA content differences between conditions (*p* < 0.05).

## 5. Conclusions

Overall, these results seem to indicate that isoprene can prime the defense systems of plants that are already under unstressed growth conditions by activating signaling pathways that help plants to better respond to drought stress and, unexpectedly, probably also to biotic stresses. This also explains why plants, where isoprene emission is suppressed, need to activate complementary defenses and secondary metabolisms.

Moreover, the proteomic analyses highlighted several proteins for which their abundance is altered in WT plants grown in stress conditions while remaining unaffected in the ISPS genotype which was grown both in control and stress conditions. This sub-group includes chloroplast proteins (involved in PSI and PSI–LHCI assembly processes; chloroplast development; tetrapyrrole biosynthesis; and the synthesis of protectants against photo-oxidative stress), proteins involved in cellular trafficking and phytosterol and BRs biosynthesis. Overall, these patterns again can be interpreted as a consequence of isoprene (a) mitigating stress occurrence and/or (b) priming a mild increase in defenses that may help readily respond to upcoming stresses. Isoprene priming of BR is a novel result that could also help explain the better photosynthetic efficiency of ISPS plants, especially after drought stress.

## Figures and Tables

**Table 1:** List of relevant differentially abundant proteins (DAPs) dependent on genotype (G), treatment (T), and interaction (I) divided according to their subcellular localization (Suba CC) and function (chloroplast-related functions, endomembrane trafficking, cellular signaling, osmoprotection, redox homeostasis, and defense). Blue and red boxes show upregulated and downregulated proteins, respectively, in the comparisons considered.

ID TAIR	Protein Description	WT DS vs. WT WW	ISPS WW vs. WT WW	ISPS DS vs. WT WW	ISPS WW vs. WT DS	ISPS WW vs. ISPS DS	ISPS DS vs. WT DS	Suba CC
GENOTYPE-DEPENDENT DAPS								
CHLOROPLAST								
AT5G10470.1	KCA1	−0.08	5.98	5.97	6.07	0.02	6.05	CYT
AT1G31800.1	CYP97A3, LUT5	0.31	6.26	6.34	5.95	−0.07	6.02	CH
AT1G53520.1	Chalcone-flavanone isomerase	0.28	4.75	4.55	4.46	0.20	4.26	CH
AT4G25100.5	FSD1	−0.07	4.09	5.79	4.16	−1.70	5.86	CH
AT5G53170.1	FTSH11	−0.03	4.40	4.22	4.43	0.18	4.25	CH
AT1G12270.1	Hop1	−0.08	4.39	4.56	4.47	−0.17	4.64	CYT
ATCG00190.1	RPOB	−0.68	4.08	4.55	4.77	−0.47	5.23	CH
ENDOMEMBRANE TRAFFICKING								
AT1G01910.4	GET3A	−0.39	7.36	7.17	7.75	0.20	7.56	CYT
AT5G16300.3	COG1	0.67	3.31	3.14	2.64	0.18	2.46	CYT,G
AT4G29160.3	SNF7.1	−0.84	5.28	5.64	6.12	−0.35	6.48	ENDO
AT5G54440.1	CLUB/TRS130	0.13	4.63	4.36	4.50	0.27	4.23	G
AT5G58030.1	TRAPPC5	0.62	6.19	5.97	5.57	0.22	5.35	G
CELLULAR SIGNALLING								
AT5G07300.1	BON2	0.34	5.04	4.69	4.70	0.35	4.35	PM
AT1G70940.1	PIN3	−0.82	4.99	4.54	5.81	0.45	5.36	PM
AT3G21220.1	MKK5	−0.30	2.83	3.05	3.13	−0.22	3.35	M
AT5G05590.1	PAI2	−0.27	5.45	5.67	5.72	−0.22	5.94	CH
AT5G09440.1	EXL4	−0.22	−5.45	−5.56	−5.23	0.11	−5.35	EXTR
TREATMENT-DEPENDENT DAPS								
AT5G23040.1	CDF1	−4.46	0.15	−4.99	4.61	5.14	−0.53	CH
AT4G04850.2	KEA3 K+ efflux antiporter 3	−5.20	−0.13	−5.56	5.07	5.43	−0.36	CH
AT5G48570.1	FKBP65	−10.96	−0.32	−10.19	10.64	9.87	0.61	CYT
AT5G16010.1	3-oxo-5-alpha-steroid 4-dehydrogenase family protein	−6.99	−0.10	−6.80	6.89	6.70	0.41	CH
AT3G02260.1	auxin transport protein (BIG)	−3.90	0.07	−3.39	3.97	3.46	−0.07	CYT
AT1G58290.1	HEMA1	4.50	0.65	5.11	−3.85	−4.46	−0.01	CH
AT3G53560.1	Tetratricopeptide repeat (TPR)-like superfamily protein	4.86	−0.16	5.26	−5.01	−5.42	0.52	CH
AT4G05090.1	Inositol monophosphatase family protein	5.81	0.48	5.74	−5.33	−5.26	0.77	CH
AT3G50360.1	CEN2	4.07	0.21	4.06	−3.87	−3.86	0.19	CYT
AT4G28470.1	RPN1B	3.69	0.16	4.21	−3.53	−4.05	0.51	CYT
INTERACTION-DEPENDENT DAPS								
ISOPRENE-SPECIFIC RESPONSE TO DROUGHT STRESS								
OSMOPROTECTANS AND REDOX HOMESOTASIS								
AT1G06410.1	trehalose-phosphatase/synthase 7	1.08	0.90	5.36	−0.18	−4.45	4.27	CYT
AT3G55610.1	P5CS2	0.52	−0.02	4.45	−0.54	−4.47	3.93	CH
AT2G26230.1	urate oxidase	0.16	0.09	3.99	−0.06	−3.89	3.83	PEROX
AT1G75270.1	DHAR2	0.03	0.33	4.25	0.30	−3.92	4.23	CYT
AT5G18100.2	copper/zinc superoxide dismutase 3	−0.61	0.25	4.30	0.87	−4.05	4.92	PEROX
AT2G22500.1	uncoupling protein 5	−0.12	0.06	4.35	0.17	−4.30	4.47	M
AT5G54430.1	PHOS32	0.46	0.43	6.17	−0.04	−5.74	5.71	CYT
AT3G13470.1	TCP-1/cpn60 chaperonin	0.03	0.24	5.11	0.21	−4.87	5.08	CH
AT1G04980.1	PDIL2-2	−0.07	−0.03	4.08	0.03	−4.11	4.14	ER
AT1G12010.1	ACO3	−0.06	0.28	5.18	0.34	−4.90	5.24	CYT
AT3G10130.1	| SOUL heme-binding	−0.15	−0.03	−2.60	0.12	2.57	−2.45	CH
AT1G21350.3	Thioredoxin	0.14	−0.03	−5.49	−0.17	5.46	−5.63	CH
DEFENSE								
AT1G02120.1	VAD1	−0.16	0.72	4.03	0.89	−3.31	4.20	ER
AT3G24550.1	PERK1	−0.78	−0.45	3.42	0.33	−3.87	4.21	PM
AT2G44490.1	PEN2	0.03	−0.20	−6.26	−0.23	6.06	−6.29	PEROX
AT5G35620.1	Eukaryotic initiation factor 4E protein	−0.29	0.12	−3.26	0.40	3.37	−2.97	N
AT1G55210.2	Disease resistance-responsive (dirigent-like protein)	0.44	−0.34	−7.38	−0.78	7.03	−7.82	EXTR
AT4G33500.1	Protein phosphatase 2C	0.62	−4.35	−4.30	−4.97	−0.05	−4.92	CH
AT3G18165.1	MOS4	0.17	3.92	4.06	3.75	−0.15	3.90	N
AT4G16990.2	RLM3	0.12	4.83	4.94	4.71	−0.11	4.82	CYT
DAPS SIMILARLY ALTERED IN WT STRESSED AND ISOPRENE-EMITTING UNSTRESSED PLANTS								
CHLOROPLAST								
AT5G48790.1	low accumulation PSII protein	6.06	5.66	5.53	−0.40	0.13	−0.53	CH
AT1G32550.1	FdC2	5.13	5.64	5.97	0.51	−0.33	0.84	CH
ATCG00150.1	ATPI	7.42	7.17	7.06	−0.25	0.11	−0.36	CH
ATCG00170.1	RPOC2	5.06	5.74	5.25	0.68	0.49	0.19	CH
CELLULAR SIGNALLING								
AT4G35860.1	RABB1B	5.81	5.52	5.69	−0.29	−0.17	−0.12	CH
AT3G44340.2	CEF	5.31	5.53	5.03	0.22	0.49	−0.28	CH
AT5G07340.1	Calreticulin	3.52	3.52	3.58	0.00	−0.05	0.05	ER
AT4G29810.1	MKK2	4.99	5.00	5.16	0.00	−0.16	0.16	PM
AT3G01090.3	KIN10	6.74	6.81	6.75	0.07	0.06	0.01	CYT
AT3G11660.1	NDR1/HIN1-like 1	5.51	5.33	5.24	−0.18	0.09	−0.27	PM
AT2G21600.1	RER1B	−3.34	−3.86	−3.85	−0.51	−0.01	−0.50	ER
AT5G65950.1	TRAPPC11	−6.27	−6.16	−6.31	0.11	0.14	−0.03	G
AT1G18210.2	Calcium-binding EF-hand family protein	−5.46	−6.30	−6.00	−0.84	−0.30	−0.54	CYT+
AT3G22370.1	alternative oxidase 1°	−5.33	−4.53	−5.13	0.79	0.59	0.20	M
AT4G23150.1	CRK7	−5.31	−4.86	−5.69	0.45	0.83	−0.38	PM
AT1G49340.2	ATPI4K ALPHA	−5.83	−5.15	−5.50	0.68	0.35	0.33	PM
AT1G76040.2	CPK29	−4.13	−4.27	−3.81	−0.14	−0.46	0.31	CYT
DAPS ALTERED BY DROUGHT STRESS IN WT BUT NOT IN TRANSGENIC ARABIDOPSIS								
CHLOROPLAST								
ATCG00520.1	YCF4	−5.40	0.05	0.33	5.45	−0.28	5.73	CH
AT3G48870.1	CLPC	−4.06	0.27	0.41	4.33	−0.14	4.47	CH
AT2G32480.1	ARASP	−5.14	0.12	0.06	5.26	0.06	5.19	CH
AT1G06820.1	CCR2 carotenoid isomerase	−4.43	−0.11	0.03	4.32	−0.14	4.45	CH
AT2G26540.1	UROS uroporphyrinogen-III synthase	−4.81	0.13	−0.13	4.94	0.26	4.68	CH
AT1G09940.1	HEMA2	7.41	0.00	−0.32	−7.42	0.32	−7.74	CH
AT1G17050.1	SPS2 solanesyl diphosphate synthase 2	5.37	−0.24	0.09	−5.61	−0.33	−5.28	CH
ATCG00360.1	YCF3	7.02	0.56	0.64	−6.46	−0.08	−6.38	CH
CELLULAR TRAFFICKING								
AT1G09210.1	CRT1b	5.54	−0.46	−0.77	−6.00	0.31	−6.31	ER
AT1G71270.1	POK Vps52 / Sac2 family	6.43	−0.19	−0.91	−6.62	0.71	−5.72	G
AT5G54750.1	Transport protein particle (TRAPP)	5.77	0.05	0.05	−5.72	0.00	5.42	G
AT1G15130.1	Endosomal targeting BRO1-like domain-containing protein	−5.47	0.07	−0.05	5.54	0.12	5.92	ENDO
AT3G25220.1	FKBP15-1	−5.59	0.33	0.33	5.92	0.00	5.78	ER
AT1G31730.1	Adaptin family protein	−5.46	0.02	0.32	5.48	−0.30	3.52	CYT,G
AT1G67930.1	Golgi transport complex protein-related	−4.00	0.02	−0.48	4.02	0.50	3.53	G
AT4G02350.1	SEC15B	−3.72	0.31	−0.19	4.03	0.50	5.40	CYT
AT3G46830.1	ATRABA2C	−5.58	−0.14	−0.17	5.44	0.03	4.87	G-ENDO
AT5G35160.1	Endomembrane protein 70 protein	−4.70	0.17	0.17	4.87	0.00	3.13	G
AT5G15970.1	KIN2	−2.89	−0.01	0.24	2.88	−0.25	6.24	CYT
AT3G48170.1	ALDH10A9	−5.35	0.86	0.89	6.22	−0.03	5.67	PEROX
AT4G01850.2	SAM2	−5.04	0.34	0.64	5.38	−0.29	11.74	CYT
AT1G35670.1	CDPK2 calcium-dependent protein kinase 2	−11.73	0.06	0.01	11.79	0.05	6.02	CYT
AT5G13710.2	SMT1	−5.89	0.33	0.14	6.22	0.20	5.68	V
AT1G20050.1	HYD1	−5.94	0.22	−0.26	6.16	0.48	−7.34	PM

## Data Availability

All data supporting the findings of this study are available within the paper and within its supplementary information published online.

## Supplementary Materials

The following supporting information can be downloaded at: https://www.mdpi.com/article/10.3390/ijms23073836/s1.
