# Supplementary figures and images for: Isoprene Emission Influences the Proteomic Profile of Arabidopsis Plants under Well-Watered and Drought-Stress Conditions
Source: Int J Mol Sci. 2022 Mar 30;23(7):3836. doi: 10.3390/ijms23073836 (PMC8998555; doi:10.3390/ijms23073836)

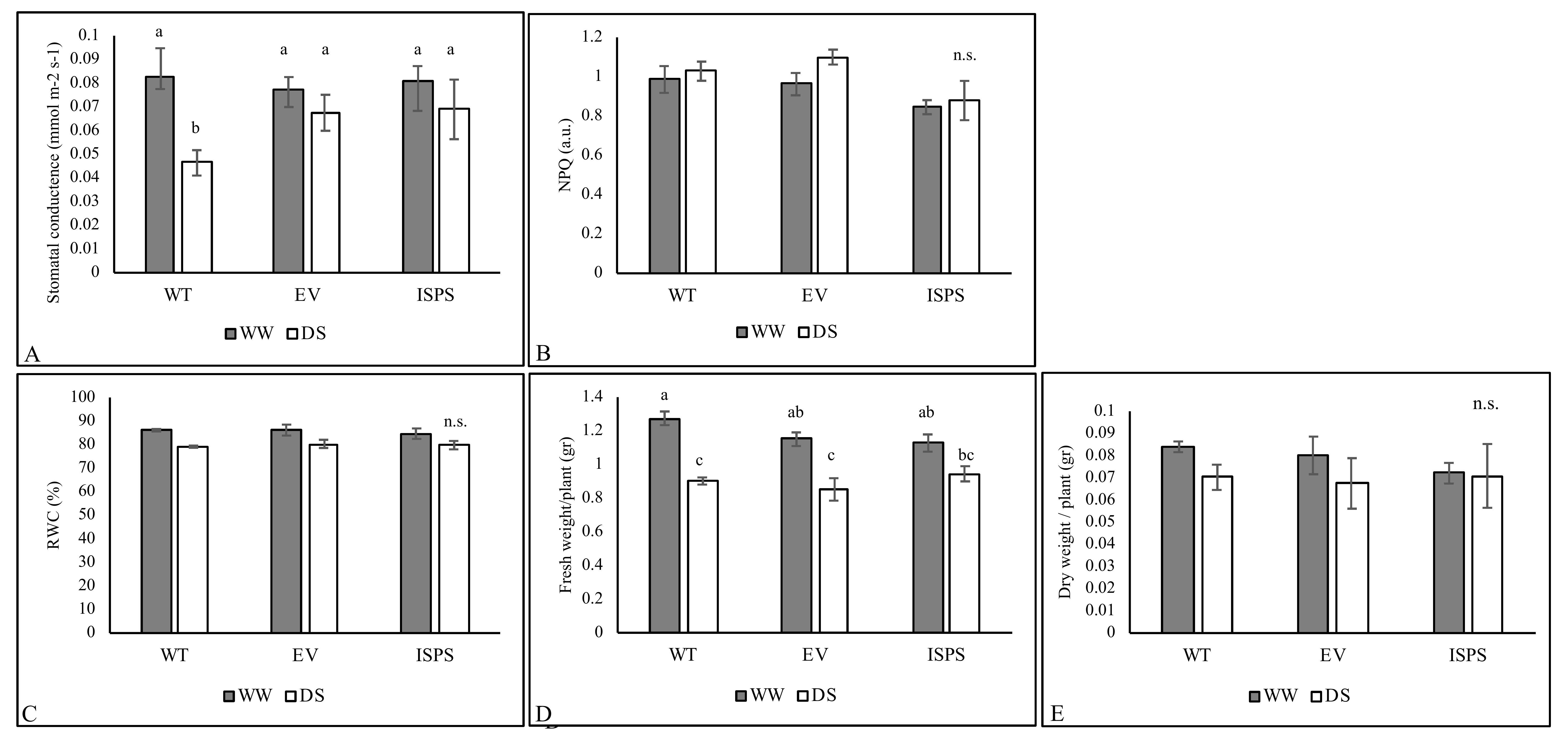

Supplement: Supplementary file 1 [file ijms-23-03836-s001.zip › Mancini et al. Supplemental materials_rev/Figure S1.jpg]

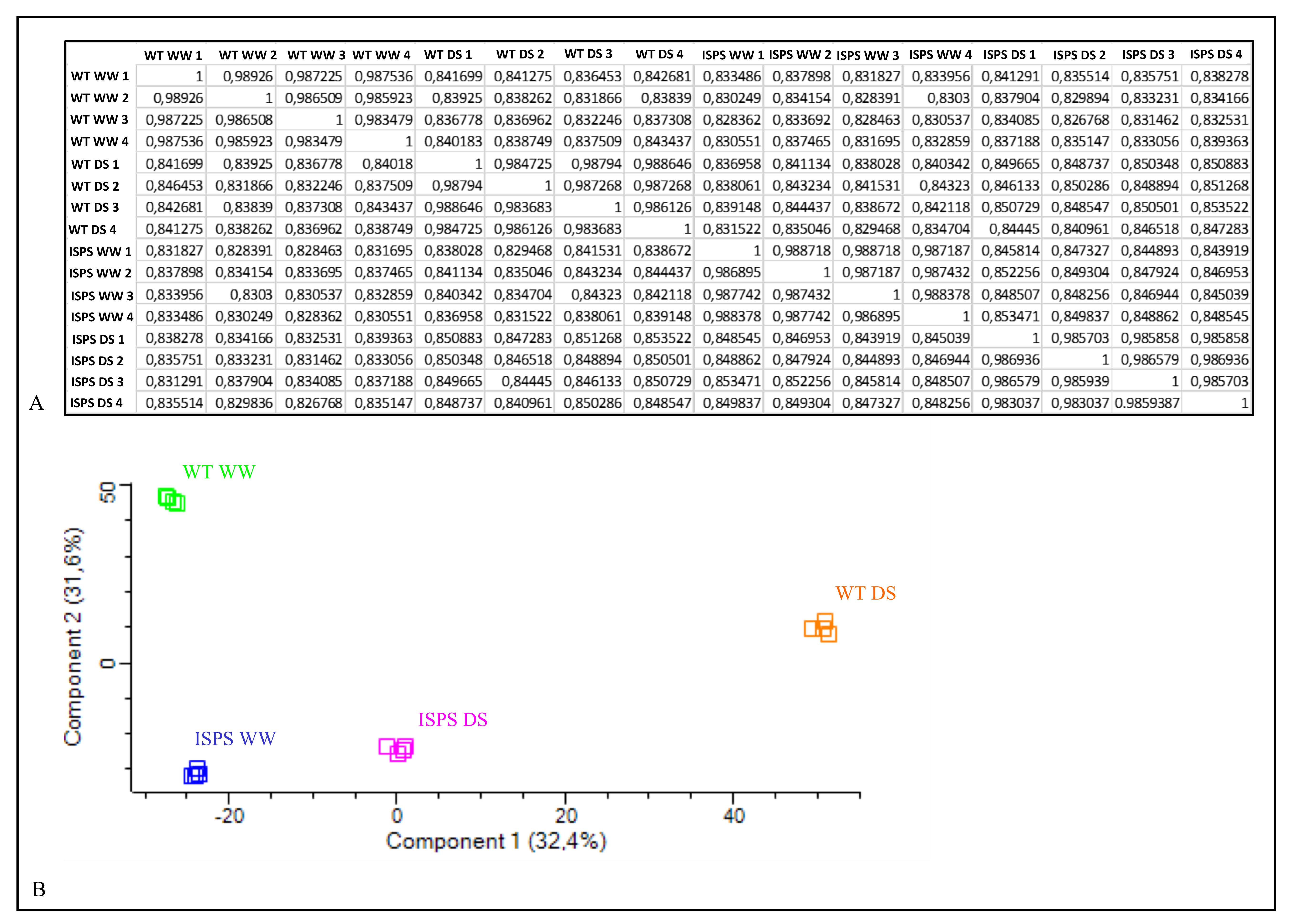

Supplement: Supplementary file 1 [file ijms-23-03836-s001.zip › Mancini et al. Supplemental materials_rev/Figure S2.jpg]
